# *Akkermansia muciniphila* Alters Gut Microbiota and Immune System to Improve Cardiovascular Diseases in Murine Model

**DOI:** 10.3389/fmicb.2022.906920

**Published:** 2022-06-14

**Authors:** Xin He, Yang Bai, Haiyang Zhou, Kemin Wu

**Affiliations:** ^1^Department of Anesthesiology, Xiangya Hospital, Central South University, Changsha, China; ^2^Department of General and Vascular Surgery, Xiangya Hospital, Central South University, Changsha, China; ^3^National Clinical Research Center for Geriatric Disorders, Xiangya Hospital, Changsha, China

**Keywords:** *Akkermansia muciniphila*, intestinal microbiology, microbial structure, abdominal aortic aneurysm, microbial physiology

## Abstract

The gut microbiota plays an important role in a variety of cardiovascular diseases. The probiotics screened based on microbiota can effectively improve metabolism and immune function of the body, which is of great value in the field of cardiovascular disease treatment. Abdominal aortic aneurysms (AAA) refer to the lesion or injury of the abdominal aortic wall resulting in a localized bulge, which is one of the cardiovascular diseases with pulsing mass as the main clinical symptom. Previous studies have confirmed that *A. muciniphila* was depleted in the guts of AAA patients or mice. *A. muciniphila* is a potential probiotic for the treatment of intestinal microbiome-related diseases. Therefore, this study aims to investigate the effects of *A. muciniphila* on gut microbiota and disease-related biomarkers in AAA mice. C57BL/6J mice were used to construct the AAA model and treated with *A. muciniphila*. Aortic aneurysm formation in the AAA group is associated with the increased diameter of the abdominal aorta and inflammatory infiltration. *A. muciniphila* inhibited the formation of AAA and repaired tissue damage. The number of gut microbiota and α diversity index were decreased in the model group. *A. muciniphila* increased the number of gut microbiota and α diversity in AAA mice. The abundance of *uncultured bacterium* and *Lactobacillus* were increased, while the abundance of the *Lachnospiraceae NK4A136 group* was reduced in the AAA group. Compared with the control group, the levels of MMP-1, MMP-9, IL-33, CTSB, and CTSL in tissue and the levels of IL-6, IFN-γ, and CRP in blood were significantly increased, and the levels of IL-4, IL-10, and IL-17A in blood were significantly decreased in the AAA group. The intervention of *A. muciniphila* reversed these changes. The gut microbiota function prediction showed changes in *E. coli, Clostridium*, and *Lactobacillus* metabolism-related functional pathways. *Akkermansia* was negatively correlated with *Helicobacter* and *Lactobacillus* and positively correlated with *Clostridium_sensu_stricto_1* and *Escherichia shigella* at the genus level. In conclusion, *A. muciniphila* inhibited the formation of AAA by restoring gut microbiota diversity, altering the expression of peripheral immune factors, and the functions of *E. coli, Clostridium*, and *Lactobacillus*, which may provide a new theoretical basis for the application of probiotics in cardiovascular diseases.

## Introduction

Abdominal aortic aneurysm (AAA) is one of many diseases associated with inflammatory cell infiltration, matrix protein degradation, and smooth muscle cell apoptosis (Liu et al., 2019). AAA is the most commonly defined as having a maximum diameter of the abdominal aorta greater than 3 cm in anteriorly or cross-section, or having a focal dilation greater than 1.5 times the diameter of a normal adjacent artery segment (Wang et al., 2018). The pathological manifestations of AAA in human and animal models showed obvious immune cell infiltration and upregulated expression of pro-inflammatory cytokines, suggesting that inflammation plays an important role in the formation of AAA (Li Y. et al., 2019; Tsai and Xian, 2019). Functional enrichment analysis showed the level of granulocyte colony-stimulating factor (G-CSF), milk fat globule-EGF factor 8 protein (MFG-E8), macrophage inflammatory protein 1 g (MIP-1g), and cardiotrophin 1 (CT-1) were positively correlated with IL-33, periostin, matrix metalloproteinase-1 (MMP-1), matrix metalloproteinase-9 (MMP-9), cathepsin B (CTSB), and cathepsin L (CTSL) in AAA mice (Li Y. et al., 2019). IL-33 is a pliotropic cytokine with multiple immunomodulatory effects (Li J. et al., 2019). Exogenous IL-33 (daily intraperitoneal administration of recombinant IL-33 or transgenic IL-33 expression) protected mice from the effects of AAA formation by enhancing ST2-dependent aortic and systemic regulatory T-cell amplification (Li J. et al., 2019). These studies suggest that these cytokines may be involved in the formation of AAA by affecting protease activity, providing a series of meaningful targets for the study of biomarkers and molecular mechanisms of AAA.

Coronary artery disease is the most common health problem worldwide and remains a leading cause for morbidity and mortality (Kazemian et al., 2020). The gut microbiota plays an important role in a variety of cardiovascular diseases, such as atherosclerosis and hypertension associated with AAA (Jie et al., 2017; Peng et al., 2018). The 16S rRNA gene sequence analysis of the gut microbiota of AAA mice revealed that the gut microbiota was different between the normal and AAA mice, and the changes of *Akkermansia, Odoribacter, Helicobacter*, and *Ruminococcus* may participate in the development of AAA (Lindskog Jonsson et al., 2018; Xie et al., 2020). The relative abundance of *A. muciniphila* in the gut of AAA mice was significantly decreased, and the diameter of the abdominal aorta was negatively correlated with *A. muciniphila* in AAA mice (Xie et al., 2020). *A. muciniphila* is a kind of intestinal symbiotic bacteria stably colonized in the intestinal mucus layer, which is considered as one of the candidate strains for the next generation of probiotics (Geerlings et al., 2018). *A. muciniphila* can effectively improve the metabolism and immune function of the body in its host (Zhang et al., 2019). *A. muciniphila* may be used as one of the new probiotics for the treatment of AAA.

Since *A. muciniphila* was first described in 2004, many studies have been conducted (Hagi and Belzer, 2021). A number of studies have shown that *A. muciniphila* is a promising target for the treatment of gut microbiota-related diseases, such as colitis, metabolic syndrome, and immune diseases (Zhou, 2017). Oral administration of *A. muciniphila* in diabetic Sprague–Dawley rats has been shown to improve liver function, reduce glucose/lipid toxicity, reduce oxidative stress, inhibit inflammation, normalize gut microbiota, and improve type 2 diabetes (Zhang et al., 2018). *A. muciniphila* combined with cisplatin (CDDP) can enhance immune regulation by regulating the differentiation of Th17 cells in mice (Chen et al., 2020). Amuc_1100, a specific protein isolated from *A. muciniphila*’s outer membrane, can interact with Toll-like receptor 2 to improve the intestinal barrier and play a probiotic role (Plovier et al., 2017). *A. muciniphila* secretes an 84 kDa protein (P9) that increases thermogenesis and glucagon-like peptide-1 (GLP-1) secretion by inducing uncoupling protein 1 in brown adipose tissue and throughout the body in high-fat diet (HFD) induced C57BL/6J mice (Yoon et al., 2021). The above studies have proved that *A. muciniphila* can regulate the gene function and intestinal homeostasis of the host through autocrine or probiotic effects and improve the disease characterization.

*Akkermansia muciniphila* ameliorates inflammation caused by metabolic endotoxemia by restoring the intestinal barrier, thereby reducing atherosclerotic lesions (Li et al., 2016). Treatment with active *A. muciniphila* reversed metabolic disorders associated with a high-fat diet, including increased fat mass, metabolic endotoxemia, adipose tissue inflammation, and insulin resistance, while heat-killing *A. muciniphila* treatment did not improve metabolic characteristics or mucous layer thickness (Everard et al., 2013). Clinical data suggest that oral administration of *A. muciniphila* is safe, but its effect needs to be further verified in a larger sample size clinical trial (Zhou, 2017; Depommier et al., 2020). However, as a new probiotic, the role of *A. muciniphila* in the occurrence of AAA disease is still unknown. In this study, *A. muciniphila* was used to treat AAA mice, and 16S rRNA gene sequence was used to analyze the gut microbiota function and cytokine expression in order to clarify the potential mechanism of *A. muciniphila* in the treatment of AAA.

## Materials and Methods

### Culture and Preparation of *A. muciniphila*

*Akkermansia muciniphila* (ATCC-BAA-865, DSM 22959) was purchased from BioVector NTCC Inc., Anaerobic culture of *A. muciniphila* (DSM 22959) in the culture medium of brain and heart infusion containing 1‰ L-cysteine for 48 h. The culture was centrifuged at 6,680 × *g* for 10 min. Then, the supernatant was discarded. The *A. muciniphila* were heavily suspended in a sterile mercaptoacetate/phosphate buffer (PBS) solution containing 25% (v/v) glycerol under anaerobic conditions. The concentration of the oral solution of *A. muciniphila* was adjusted to 1 × 10^8^ CFU mL^–1^, and the same concentration of the oral solution was inactivated by pasteurization at 70°C for 30 min. Bacterial concentrations were determined by plate counting using brain and heart extract agar (Land Bridge Technology Corporation, Beijing, China). Inactivated *A. muciniphila* suspension was stored at –80°C.

### Animal Experiments and Grouping

**FIGURE 1:**
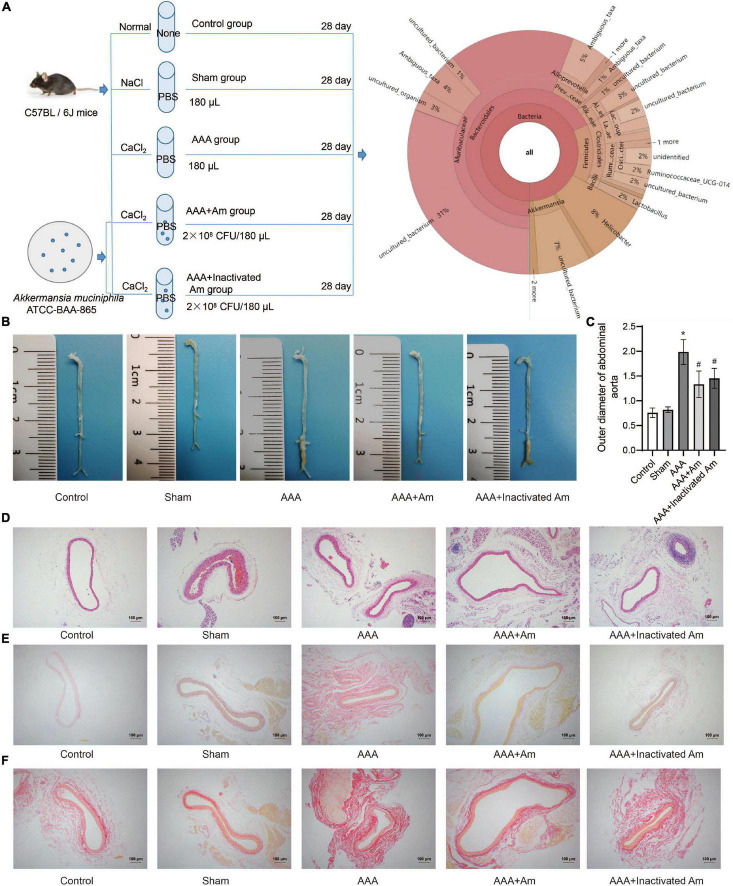
*A. muciniphila* inhibit the formation of AAA. **(A)** The experimental design flowchart. **(B)** The distribution of abdominal aortic aneurysms in mice under different treatments. **(C)** The abdominal aortic dilatation degree of rats in different treatment groups. **(D)** HE staining was used to observe the pathological changes of abdominal aorta. **(E,F)** The distribution of collagen fibers was observed by picrosirius red staining and Van Gieson staining. Data were shown as means ± SD and analyzed by two-way ANOVA (*n* = 10 mice/group). *Compared with the control group, *P* < 0.05; ^#^compared with the AAA group, *P* < 0.05.

A total of 50 male C57BL/6J mice aged 5–6 months were used in this study. All mice were kept in cages and raised in a temperature-controlled room at 22–26°C with relative humidity of 45%–60% and standard food and water on a 12/12 h light and dark cycle. Mice were randomly divided into five groups, including the control group, sham group, AAA group, AAA + Am group, and AAA + Inactivated Am group, 10/group/squirrel cage. The mice in the AAA group and sham group were anesthetized by intraperitoneal injection of pentobarbital (40 mg kg^–1^), and induced by CaCl_2_ and NaCl (0.9%), respectively (Zhong et al., 2019). The mice in the AAA + Am group and AAA + Inactivated Am group were given 2 × 10^8^ CFU/180 μl of *A. muciniphila* by oral gavage. The mice in the AAA group and sham group were given the same dose of sterile PBS by oral gavage. There was no treatment in the control group. On the 28th day of the experiment, fecal samples were collected and stored at –80°C. The experimental design flowchart is shown in the Figure 1A. Of note, 5 fecal samples were collected from each group in the squirrel cage with 10 mice/group. Mice were sacrificed with an overdose of pentobarbital (500 mg kg^–1^), then 0.9% normal saline was injected into the left ventricle at a slow and uniform rate, and the right atrial appendage was cut. The blood flowed out of the right atrial appendage until no blood color was found. The PBS injection was stopped, and the aorta (from the aortic arch to the iliac abdominal aorta bifurcation) was taken. The aorta was dissected under an anatomical microscope (the aorta was placed in a petri dish filled with 0.9% normal saline). Fat and connective tissues attached to the outer membrane of the aorta were removed. The aorta was photographed, and the diameter of the abdominal aorta was measured.

### Abdominal Aortic Aneurysms Model Construction

Male C57BL/6J mice were anesthetized by an intraperitoneal injection of pentobarbital (40 mg kg^–1^) before laparotomy. The abdominal aortic passages below the renal artery and above the bifurcation of the artery were isolated from the surrounding retroperitoneal structures. Then, cotton gauze containing 0.5 mol L^–1^ CaCl_2_ was spread on the outer surface of the aortic passage for 15 min. The aorta was then rinsed with 0.9% sterile saline and the incision was closed. All model mice were euthanized 28 days after CaCl_2_ stimulation under different treatment groups.

### 16S rRNA Gene Sequencing and Analysis

On the 28th day of the experiment, fecal samples were collected and stored at –80°C. DNA was extracted from 200 mg fecal sample using the fecal genomic DNA extraction kit (tiangen.cat.# dp328-02) as per the manufacturer’s instructions. The dsDNA HS Assay Kit for Qubit (Shanghai Yishen Biotechnology Co., Ltd., CAT.12640ES76) was used for concentration detection. Phusion enzyme (ApexBio K1031) and primers in the V3-V4 region of the 16S rRNA gene (357F 5′-ACTCCTACGGRAGGCAGCAG-3′ and 806R 5′-GGACTACHVGGGTWTCTCATAT-3′) were used for PCR amplification and adaptor addition. The magnetic beads were sorted using BMSX-200 kit (Wuxi Baimag Biotechnology Co., Ltd.). Agarose Gel DNA Recovery Kit (Tiangen.cat.# dp209-03) was used for DNA recovery. Mixed samples were sequenced by Illumina NovaseQ6000 PE250 to obtain raw data. QIIME 2 (version 2020.2) and DADA2 were used for quality control of raw data to obtain clean data that could be used for subsequent analysis (Caporaso et al., 2010). Qiime2 was used to train the classifier in the silva-132-99 representative sequence database for species annotation and representative sequence alignment for each ASV/OTU sequence (Pruesse et al., 2007). QIIME 2 (version 2020.2) and R software (version 4.0.2) were used for sequence data analysis (Caporaso et al., 2010). QIIME2 software was used to calculate the alpha diversity index (e.g., Chao1, ACE, Shannon, and Simpson evenness index) and draw the ranked abundance curve based on ASV and a dilution curve based on alpha diversity. R software (version 4.0.2) was used to draw the histogram of relative species abundance (R ggplot2 package), the heatmap of genus abundance (R reshape2/ggplot2 package), the box plot of alpha diversity difference (R phyloseq package), the principal component analysis (PCA) and non-metric multidimensional scaling (NMDS) based on the Bray-Curtis distance (phyloseq/vegan package), and the phylogenetic diversity analysis based on the Wald test (R DESeq2 package). Venn diagrams first used R software (Venn diagram package) to generate the list of ASVs owned by samples or groups and then used jvenn^1^ web pages to visualize common and specific ASVs between samples or groups. Phylogenetic investigation of communities by reconstruction of unobserved states (PICRUSt^2^) and the MetaCyc database^3^ were used for microbial function prediction (Klindworth et al., 2013). Linear discriminant analysis (LDA) effect size (LefSe^4^) was used to analyze the MetaCyc pathways (Li et al., 2020).

### ELISA Assay

The samples of abdominal aorta tissues were collected and washed with precooled PBS (0.02 mol L^–1^, pH 7.0–7.2). The abdominal aorta tissues were cut into small pieces and put into a tissue grinder (homogenate tube). Then, 500 μl PBS was added to prepare a homogenate. The tissue homogenate was freeze-thawed 1–2 times overnight and centrifuged at 5,000 × *g* at 2–8°C for 5 min to get the supernatant. The whole blood samples were placed at room temperature for 2 h, centrifuged at 1,000 × *g* at 2–8°C for 15 min. After that, the supernatant was taken for detection. According to the manufacturer’s instructions, the levels of MMP-1 (ml037721, Shanghai Meilian, Shanghai, China), MMP-9 (CSB-E08007m, CUSABIO, Wuhan, China), CTSB (CSB-EL006185MO, CUSABIO, Wuhan, China), CTSL (E-EL-M0251c, Elabscience, Wuhan, China), IL-4 (CSB-E04634m, CUSABIO, Wuhan, China), IL-6 (CSB-E04639m, CUSABIO, Wuhan, China), IL-10 (CSB-E04594m, CUSABIO, Wuhan, China), IL-17A (CSB-E04608m, CUSABIO, Wuhan, China), IFN-γ (CSB-E04578m, CUSABIO, Wuhan, China), and C-reactive protein (CRP, CSB-E07922r, CUSABIO, Wuhan, China) were analyzed by ELISA assay.

### Hematoxylin-Eosin Staining

The abdominal aortic tissue samples of mice were taken and fixed in 4% paraformaldehyde for 24 h, followed by gradient dehydration with 20% and 30% sucrose solutions. The abdominal aortic tissue was sliced, dehydrated, embedded in paraffin, successively sliced with a paraffin slicer, connected to the treated glass slides, and baked at 60°C for 12 h. The slices were placed in xylene for 20 min × 3 times. Then, the slices were placed in 100%, 95%, 85%, and 75% ethanol successively for 5 min at each stage for dewaxing to water. Hematoxylin (Wellbio, Changsha, China) was dyed for 3 min, washed with distilled water, and PBS was returned to blue. The slices were dyed with eosin (Wellbio, Changsha, China) for 5 s and rinsed with distilled water. The slices were soaked in gradient alcohol (95–100%) and dehydrated for 5 min per grade. After removal, the slices were placed in xylene for 10 min × 2 times, sealed with neutral gum, and observed under a microscope (BA210T, Motic).

### Picrosirius Red and Van Gieson Staining

The slices were baked at 160°C for 12 h. The slices were placed in xylene for 20 min × 3 times. Then, the slices were placed in 100, 95, 85, and 75% ethanol successively for 5 min at each stage for dewaxing to water. Picrosirius red staining solution (Wellbio, Changsha, China) was dyed for 10 min. After that, the slices were rinsed with distilled water and then rinsed with running water for 5 s. Van Gieson staining solution (Wellbio, Changsha, China) was also dyed for 2 min. After that, the slices were rinsed with distilled water and then with tap water for 5 s. The slices were soaked with gradient alcohol (95–100%) and dehydrated for 5 min per grade. After removal, the slices were placed in xylene for 10 min × 2 times, sealed with neutral gum, and observed under a microscope (BA210T, Motic).

### Immunohistochemistry

The slices were immersed in 0.01 M citrate buffer (pH 6.0), heated continuously in an electric furnace or microwave oven for 20 min for thermal repair of antigen. The endogenous enzyme was inactivated by adding 1% periodate acid at room temperature for 10 min. Primary antibody IL-33 (12372-1-AP, 1:200, Proteintech, United States) was incubated overnight at 4°C, followed by the secondary antibody anti-rabbit-IgG-HRP polymer at 37°C. Finally, the slices were visualized with diaminobenzidine (DAB, ZSGB-Bio, Peking, China) substrate, re-dyed with hematoxylin, gradient dehydration with all levels of alcohol (60–100%), neutral gum seal, and microscopically observed. The IPP (Image-Pro-Plus) software was used for image analysis and processing.

### Real-Time Reverse Transcription-PCR Assay

The abdominal aorta tissues of mice in different treatment groups were collected. Total RNA in tissues was extracted by Trizol reagent (Thermo Fisher Scientific, MA, United States). The Hifiscript cDNA Synthesis Kit (Covin Biosciences, Changsha, China) was used for cDNA synthesis. Then quantitative real-time PCR was carried out by using an UltraSYBR mixture (Covin Biosciences, Changsha, China). The primers for IL-33 were designed by using Primer 5 software after searching for the target gene mRNA sequences on NCBI. The sequences for primers were listed as follows: IL-33, sense 5′-ATTCTTGGCTTACGATGTTGT-3′; antisense 5′-TCCTTCAGTTTCTTTACCAACGC-3′. GAPDH, sense 5′-GC GACTTCAACAGCAACTCCC-3′; antisense 5′-CACCCTGTT GCTGTAGCCGTA-3′. GAPDH was used as an internal control to calculate the target genes expression by using the 2^–ΔΔ*Ct*^ method.

### Western Blot

After the experiment, the abdominal aorta tissues of mice in different treatment groups were collected. The proteins were extracted and determined by radioimmunoprecipitation analysis (RIPA), lysis buffer, and BCA (bicinchoninic acid) method. A total of 200 μg protein samples were separated by 12% sodium dodecyl sulfate polyacrylamide gel electrophoresis (SDS-PAGE). The isolated proteins were transferred to a polyvinylidene fluoride membrane that had been activated by methanol and blocked by 5% skim milk, and dried at room temperature for at least 1 h. Then, membranes were incubated with primary antibodies anti-IL-33 (1:1,000, ab187060, Abcam, United States), anti-PCNA (1:5,000, 10205-2-AP, proteintech, United States), anti-OPN (1:2,000, 22952-1-AP, proteintech, United States), anti-α-SMA (1:1,000, 14395-1-AP, proteintech, United States), and anti-β-actin (1:5,000, 60008-1-Ig, Proteintech, United States) for overnight at 4°C. After being washed with cold TBST, the membranes were incubated with horseradish peroxidase-conjugated secondary antibodies anti-IgG (1:5,000, SA00001-1; 1:6,000, SA00001-2, Proteintech, United States). Finally, the specific proteins were visualized with enhanced chemiluminescence detection reagent (Thermofisher, United States). The quantification was performed by dividing the intensity of each target protein with the intensity of total β-actin on the blot.

### Analysis and Statistics

The statistical software SPSS 21.0 (IBM, United States) was used to analyze the data in this study. All the data were presented as means ± SD. Statistical significance between two groups within experiments was determined by unpaired two-tailed Student’s *t*-tests and among more than two groups using ANOVA; *N* = 5 samples/group; *P*-value < 0.05 was considered statistically significant.

## Results

### *Akkermansia muciniphila* Inhibited the Formation of Abdominal Aortic Aneurysms in Mice

The experimental design flowchart is shown in Figure 1A. After the experiment, the observation of aortic aneurysm showed that compared with the control group, aortic aneurysm was formed in the AAA group, indicating that the model construction was successful (Figure 1B). Compared with the AAA group, the distribution of aortic aneurysm was reduced in the AAA + Am and AAA + Inactivated Am groups, indicating that *A. muciniphila* inhibited the formation of aortic aneurysm in AAA mice (Figure 1B). The measurement of the maximum diameter of the abdominal aorta showed that the AAA group had the largest diameter, and the diameter of the abdominal aorta decreased significantly after *A. muciniphila* intervention (Figure 1C). Hematoxylin-eosin (HE) staining showed thinning of the middle membrane of the abdominal aorta tissue and an obvious accumulation of inflammatory cells in the middle and outer membrane in AAA mice (Figure 1D). Picrosirius red and van Gieson staining showed that the elastic fibers of the vascular wall were degraded, continuity was interrupted, and a large number of inflammatory cells invaded the septum (Figures 1E,F). The intervention of *A. muciniphila* improved the injury of elastic fibers in the vascular wall, inhibited the infiltration of inflammatory cells, and protected the integrity of the septum media in the abdominal aorta of AAA mice (Figures 1D–F). The above results indicated that *A. muciniphila* could inhibit the formation of aortic aneurysms in AAA mice.

### *Akkermansia muciniphila* Affected the Diversity and Composition of the Gut Microbiota in Abdominal Aortic Aneurysms Mice

**FIGURE 2:**
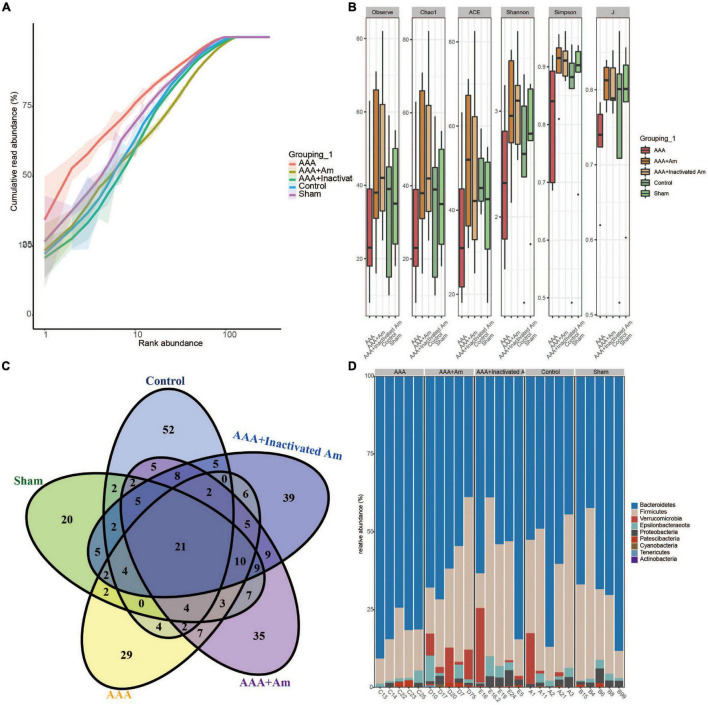
*A. muciniphila* affects the diversity of gut microbiota. **(A)** Rank abundance curve was used to evaluate sequencing depth. **(B)** The α diversity analysis of gut microbiota. **(C)** Venn diagram showed changes in the number of microbiota in the gut. **(D)** The relative abundance of gut microbiota at the phylum level.

**FIGURE 3:**
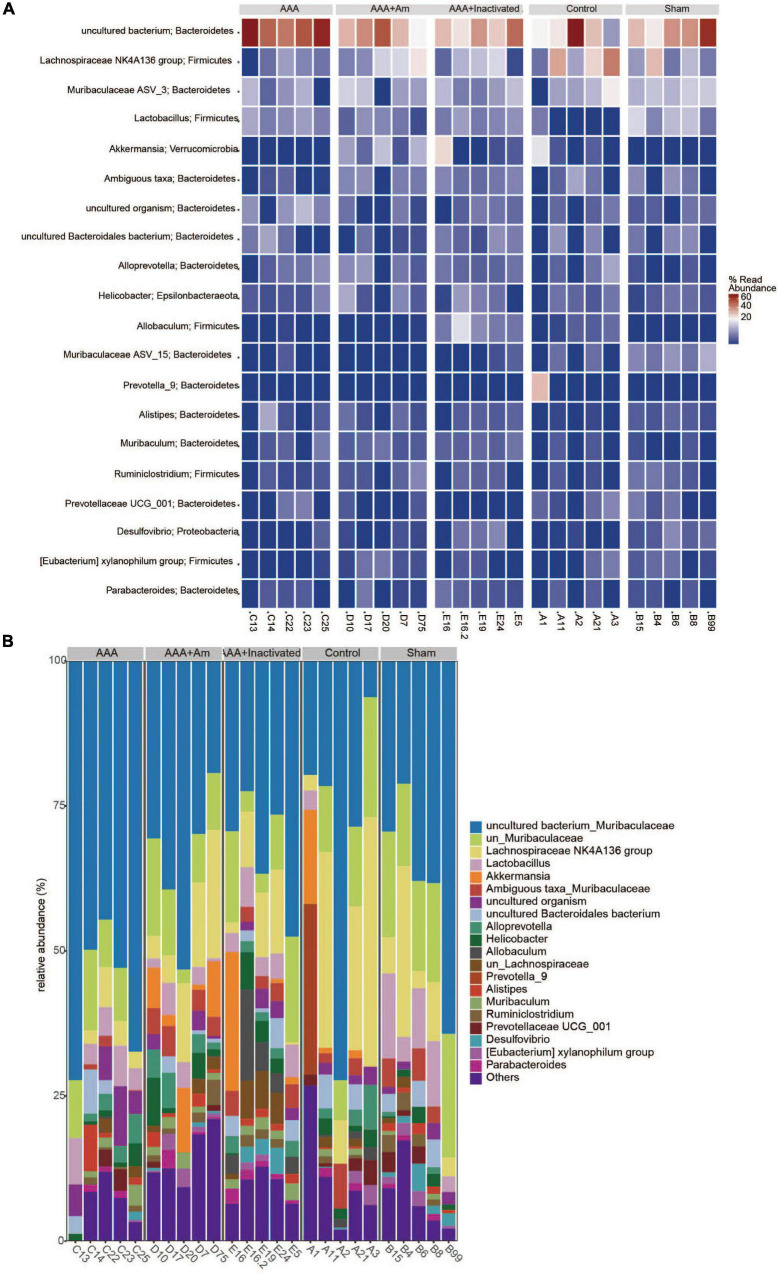
*A. muciniphila* affects the composition of gut microbiota. **(A)** Heatmap showed the relative abundance of top 20 genera in gut. **(B)** The relative abundance of gut microbiota at the genus level (*n* = 5 samples, 10 mice/group).

The rank abundance curve showed that with the increase of sequencing depth, the read abundance of each sample increased exponentially and reached the plateau stage, indicating that the sequencing depth was sufficient (Figure 2A). Based on the analysis of α diversity index, compared with the control group, the microbial diversity of gut microbiota in the AAA group was decreased (Figure 2B). The intervention of *A. muciniphila* increased the α diversity index of gut microbiota in AAA mice (Figure 2B). Venn plot analysis showed 118, 98, 101, 134, and 132 microbiota were annotated in the control group, sham group, AAA group, AAA + Am group, and AAA + Inactivated Am group, respectively (Figure 2C). The Bacteroidetes, Firmicutes, Verrucomicrobia, Epsilonbacteraeota, Proteobacteria, Patescibacteria, Cyanobacteria, Tenericutes, and Actinobacteria were annotated at phylum level (Figure 2D). The heatmap showed the top 20 genera-phylum, which mainly belong to Bacteroidetes, Verrucomicrobia, Firmicutes, and Proteobacteria (Figure 3A). The *Lachnospiraceae NK4A136 group, Lactobacillus, Akkermansia, Alloprevotella, Helicobacter, Allobaculum, Prevotella_9, Alistipes, Muribaculum, Ruminiclostridium, Prevotellaceae UCG_001, Desulfovibrio, [Eubacterium] xylanophilum group*, and *Parabacteroides* were annotated at the genus level (Figure 3B). The results showed that the intervention of *A. muciniphila* increased the diversity of gut microbiota in AAA mice.

### *Akkermansia muciniphila* Changed the Gut Microbiota Structure

**FIGURE 4:**
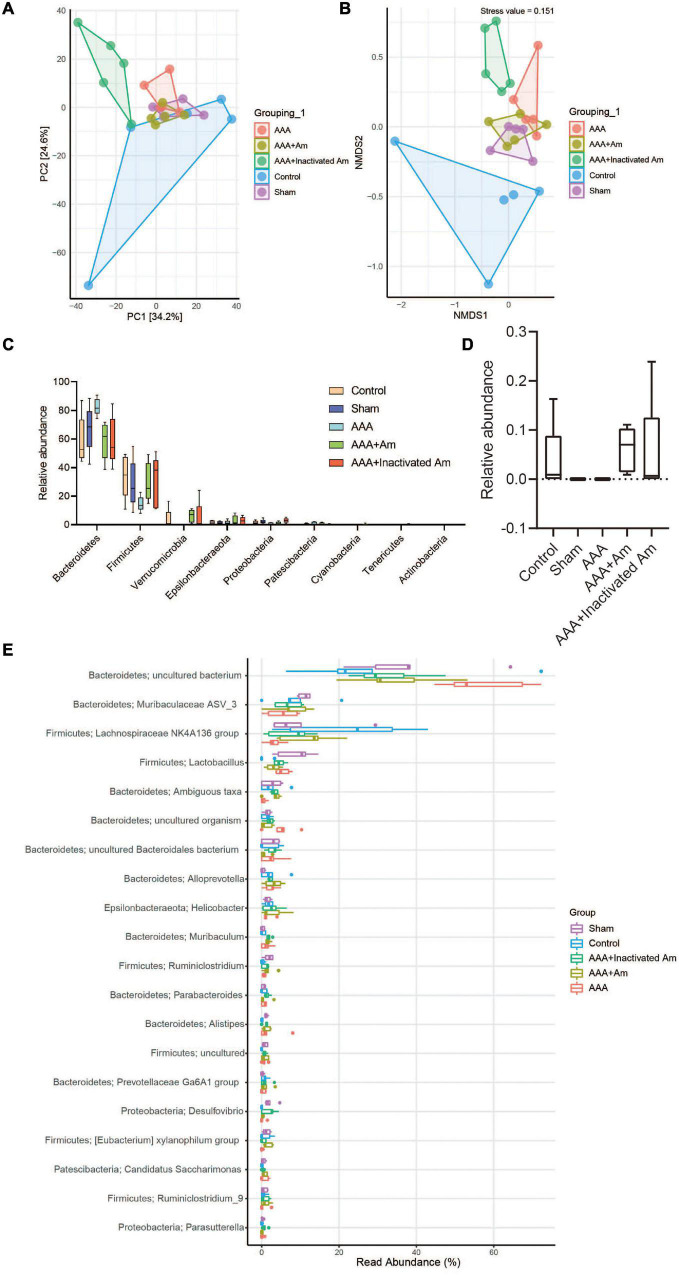
*A. muciniphila* alters the structure of gut microbiota. **(A)** PCA was used to analyze the diversity of gut microbiota. **(B)** NMDS was used to analyze the differences between groups. **(C)** The abundance of gut microbiota changes at the phylum level. **(D)** The relative abundance of *Akkermansia* at the genus level. **(E)** The relative abundance of gut microbiota changes at the genus level (*n* = 5 samples, 10 mice/group).

A principal component analysis (PCA) showed PC1 = 34.2% and PC2 = 24.6%, indicating that *A. muciniphila* explained 58.8% of the difference in microbiota composition (Figure 4A). The non-metric multidimensional scaling (NMDS) analysis showed that the stress value is equal to 0.151 and less than 0.2, and the degree of difference between groups was significant (Figure 4B). Compared with the control group, Bacteroidetes and Patescibacteria were increased in the AAA group, whereas Firmicutes, Verrucomicrobia, and Proteobacteria were decreased (Figure 4C). Compared with the AAA group, the abundance of Bacteroidetes and Patescibacteria was decreased, while the abundance of Firmicutes and Verrucomicrobia was increased in the AAA + Am group (Figure 4C). The results of the AAA + Inactivated Am group were consistent with those of the AAA + Am group (Figure 4C). Compared with the control group, the relative abundance of *Akkermansia* was decreased in the AAA group at the genus level (Figure 4D). The intervention of *A. muciniphila* improved the relative abundance of *Akkermansia* in the AAA + Am and AAA + Inactivated Am groups (Figure 4D). Bacteroidetes-*uncultured bacterium* and Firmicutes-*Lactobacillus* abundances were increased, while Firmicutes–*Lachnospiraceae NK4A136 group* abundance was decreased in the AAA group compared to the control group (Figure 4E). Bacteroidetes-*uncultured bacterium* and Firmicutes-*Lactobacillus* abundances were decreased, while Firmicutes–*Lachnospiraceae NK4A136 group* abundance was increased after being treated with *A. muciniphila* in the AAA mice (Figure 4E). A. muciniphila changed the structure of gut microbiota and might be associated with the abundance of uncultured bacterium, Lactobacillus, and Lachnospiraceae NK4A136 group.

### *Akkermansia muciniphila* Regulated Immune Factors’ Expression in Abdominal Aortic Aneurysms Mice

**FIGURE 5:**
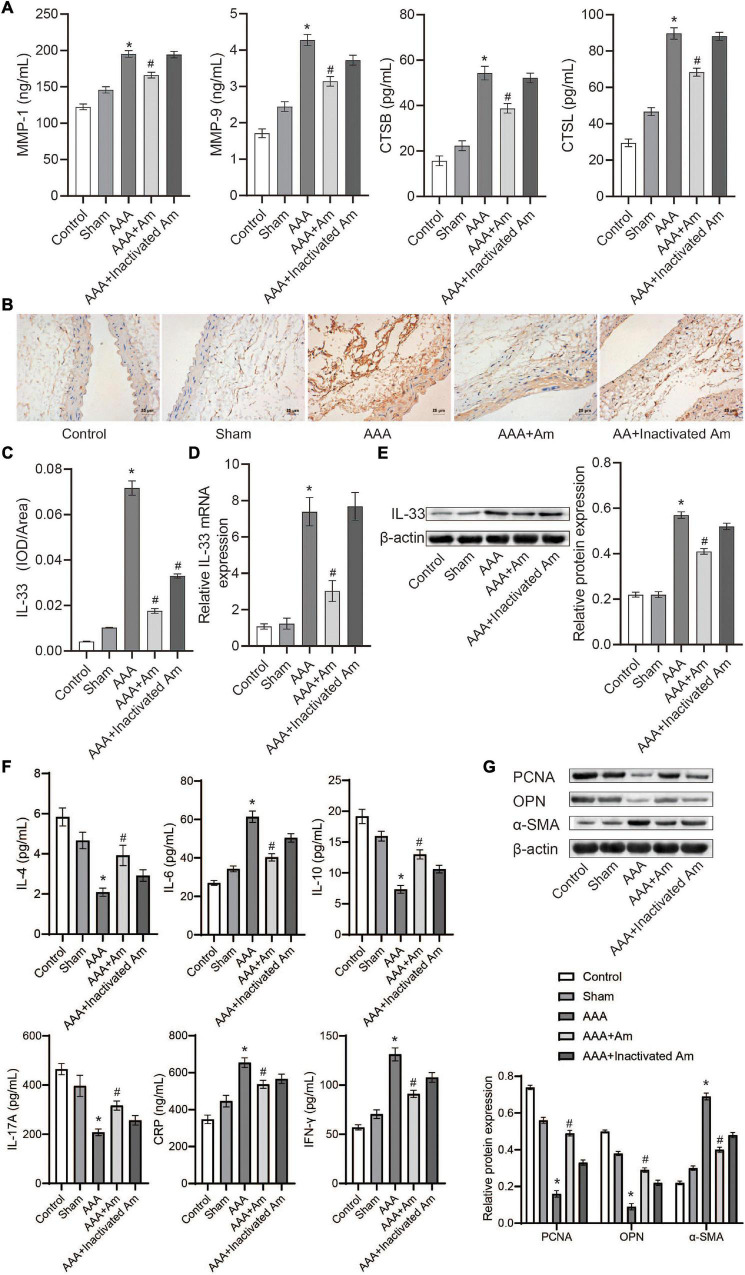
*A. muciniphila* regulates the expression of immune factors. **(A)** The levels of MMP-1, MMP-9, CTSB, and CTSL in abdominal aortic tissue were analyzed by ELISA. **(B,C)** The expression of IL-33 in abdominal aortic tissue was analyzed by IHC (scale bar = 25 μm; the magnification is 400×). **(D)** qRT-PCR was used to analyze IL-33 gene expression in abdominal aortic tissue. **(E)** The expression of IL-33 protein in abdominal aortic tissue was analyzed by Western blot. **(F)** The levels of IL-4, IL-6, IL-10, IL-17A, IFN-γ, and C-reactive protein (CRP) in peripheral blood were determined by ELISA. **(G)** The expression of PCNA, OPN, and α-SMA protein in abdominal aortic aneurysm tissue were detected by Western blot. Data were shown as means ± SD and analyzed by two-way ANOVA (*n* = 10 mice/group). *Compared with the control group, *P* < 0.05; ^#^compared with the AAA group, *P* < 0.05.

Compared with the control group, the levels of MMP-1, MMP-9, CTSB, and CTSL in the abdominal aorta tissue of the AAA group were significantly increased (Figure 5A). After the intervention of *A. muciniphila*, the levels of MMP-1, MMP-9, CTSB, and CTSL were significantly decreased in the abdominal aorta tissue (Figure 5A). Immunohistochemistry (IHC) analysis of the distribution of IL-33 in the abdominal aorta tissue showed that compared with the control group, the expression of IL-33 was significantly increased in the AAA group (Figures 5B,C). After the intervention of *A. muciniphila*, the expression of IL-33 was significantly decreased compared to the AAA group (Figures 5B,C). The results of IL-33 expression were analyzed by real-time reverse transcription-PCR assay (qRT-PCR) and Western blot in the abdominal aorta tissue were consistent with those of IHC (Figures 5D,E). ELISA was used to detect inflammatory factors in peripheral blood, and the results showed that compared with the control group, the levels of IL-4, IL-10, and IL-17A were significantly decreased, while the levels of IL-6, IFN-γ, and CRP were significantly increased in the AAA group (Figure 5F). After the intervention of *A. muciniphila*, the levels of IL-4, IL-10, and IL-17A were significantly increased, while the levels of IL-6, IFN-γ, and CRP were significantly decreased compared to the AAA group (Figure 5F). Compared with the control group, the expression of PCNA and OPN was significantly decreased, while the expression of α-SMA was significantly increased in the AAA group (Figure 5G). Compared with the AAA group, the expression of PCNA and OPN was significantly increased, while the expression of α-SMA was significantly decreased after treated with *A. muciniphila* in the AAA mice (Figure 5G). It was proved that *A. muciniphila* might inhibit the formation of AAA by regulating the expression of IL-33 and immune factors in the abdominal aorta of AAA mice.

### *Akkermansia muciniphila* Regulated the Function of Gut Microbiota to Inhibit the Formation of Abdominal Aortic Aneurysms

**FIGURE 6:**
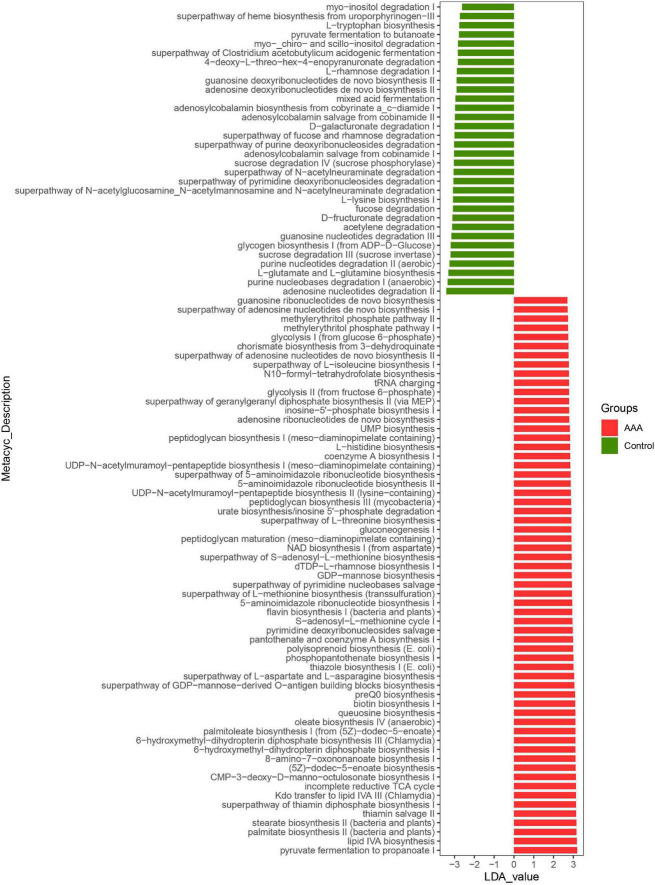
LefSe analyzed the effect of *A. muciniphila* on gut microbiota function in the control group and AAA group. The red and green represented the AAA group and control group, respectively. The prediction of gene function profile using the PICRUSt2 software and MetaCyc database. Data were analyzed by LefSe (*n* = 5 samples, 10 mice/group).

**FIGURE 7:**
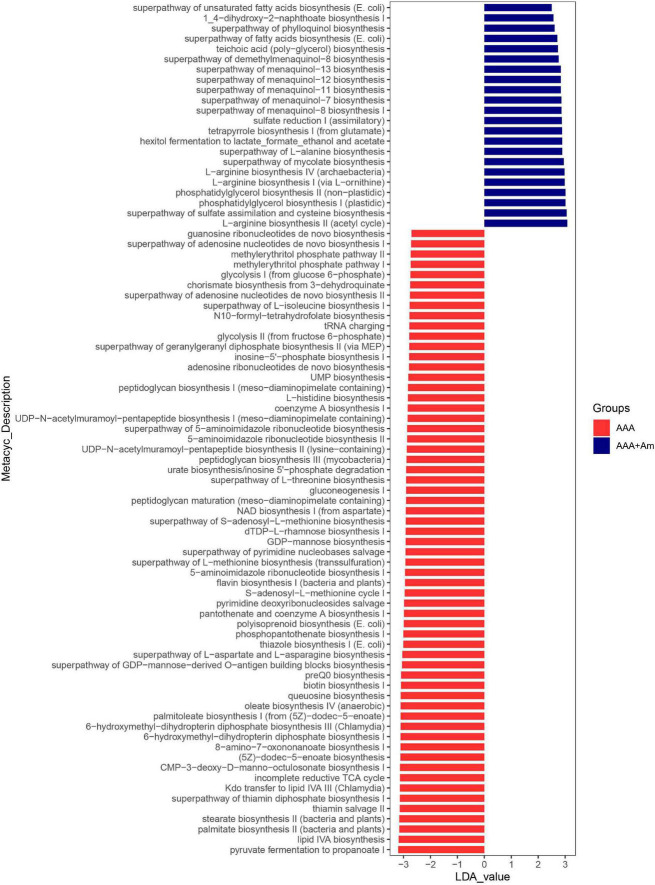
LefSe analyzed the effect of *A. muciniphila* on gut microbiota function in the AAA group and AAA + Am group. The red and blue represented the AAA group and AAA + Am group, respectively. The prediction of gene function profile using the PICRUSt2 software and MetaCyc database. Data were analyzed by LefSe (*n* = 5 samples, 10 mice/group).

*Akkermansia muciniphila* could regulate the structure of the intestinal flora of AAA mice and the expression of IL-33 in the abdominal aorta tissues, but its functional impact on the intestinal flora was not yet known. Based on the lefSe analysis, it was found that some signal pathways related to bacterial metabolism were significantly enriched in the control group, AAA group, and AAA + Am group (*P* < 0.05, LDA ≥ 2.5). The palmitate biosynthesis II (bacteria and plants, LDA = 3.15), stearate biosynthesis II (bacteria and plants, LDA = 3.15), Kdo transfer to lipid IVA III (*Chlamydia*, LDA = 3.13), 6-hydroxymethyl-dihydropterin diphosphate biosynthesis III (*Chlamydia*, LDA = 3.10), thiazole biosynthesis I (*E. coli*, LDA = 3.00), polyisoprenoid biosynthesis (*E. coli*, LDA = 2.99), flavin biosynthesis I (bacteria and plants, LDA = 2.94), peptidoglycan biosynthesis III (*mycobacteria*, LDA = 2.88), and dTDP-L-rhamnose biosynthesis I (LDA = 2.91) were significantly enriched in the AAA group (Revised Figures 6, 7).

**FIGURE 8:**
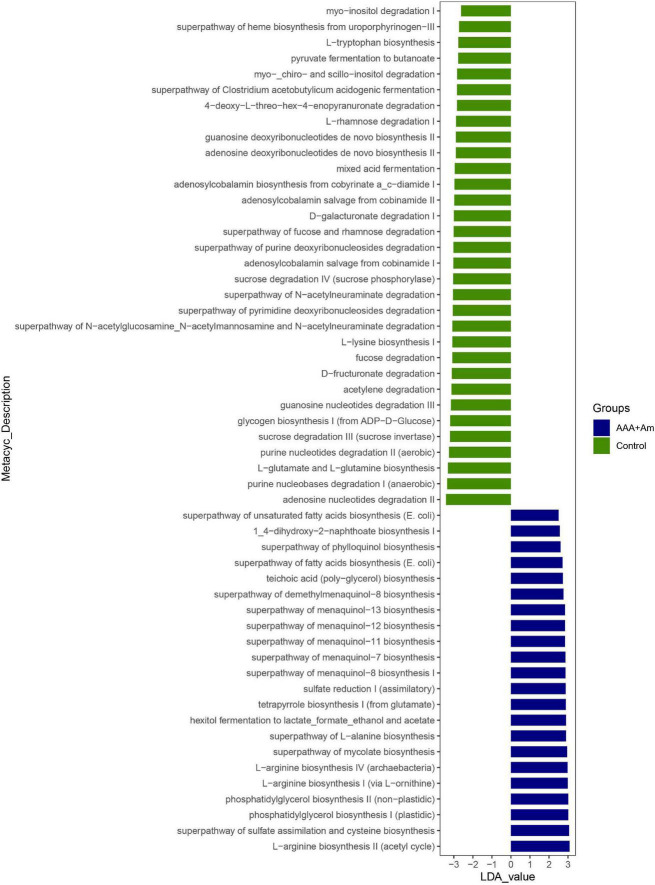
LefSe analyzed the effect of *A. muciniphila* on gut microbiota function in the Control group and AAA + Am group. The green and blue represented the control group and AAA + Am group, respectively. The prediction of gene function profile using the PICRUSt2 software and MetaCyc database. Data were analyzed by LefSe (*n* = 5 samples, 10 mice/group).

The L-arginine biosynthesis IV (*archaebacteria*, LDA = 2.97), superpathway of mycolate biosynthesis (LDA = 2.94), superpathway of fatty acid biosynthesis (*E. coli*, LDA = 2.71), and superpathway of unsaturated fatty acid biosynthesis (*E. coli*, LDA = 2.50) pathways were significantly enriched in the AAA + Am group (Revised Figures 7, 8). The superpathway of Clostridium acetobutylicum acidogenic fermentation (LDA = 2.83), superpathway of fucose and rhamnose degradation (LDA = 3.00), and L-rhamnose degradation I (LDA = 2.88) pathways were significantly enriched in the control group (Revised Figures 6, 8). These results suggest that a variety of microbial-related metabolic pathways are enriched in abdominal aortic aneurysm disease and treatment, or the potential mechanism of *A. muciniphila*’s inhibition of AAA formation through distal regulation of intestinal microorganisms.

### The Correlation Between *Akkermansia* and Gut Microbiota at Genus Level

**FIGURE 9:**
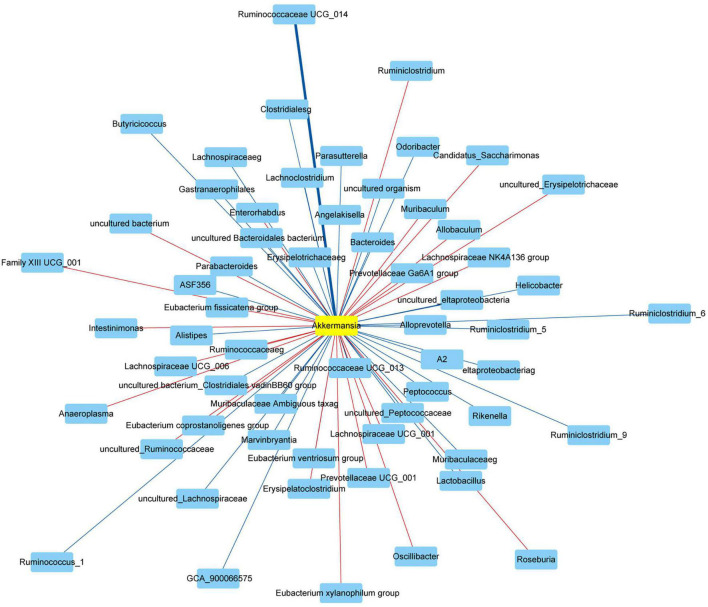
Network showed the correlation between *Akkermansia* and gut microbiota. The red and blue represented the positive and negative correlation with *Akkermansia* at the genus level, respectively. Data were analyzed using Spearman’s correlation (*n* = 5 samples, 10 mice/group).

Finally, we used the Spearman’s correlation to analyze the correlation between *Akkermansia* and gut microbiota at the genus level. The *Akkermansia* was positively correlated with *Bifidobacterium, Enterorhabdus, Bacteroides, Muribaculum, Alloprevotella, Prevotella_9, Prevotellaceae Ga6A1 group, Rikenella, Parabacteroides, Clostridium sensu stricto_1, Family XIII UCG_001, Eubacterium nodatum group, ASF356, Anaerostipes, Peptococcus, Coprococcus_2, Lachnoclostridium, Lachnospiraceae NC2004 group, Roseburia, Lachnospiraceae NK4A136 group, Marvinbryantia, Lachnospiraceae UCG_001, Eubacterium fissicatena group, Eubacterium ventriosum group, Romboutsia, Intestinimonas, Oscillibacter, Ruminiclostridium_5, Ruminiclostridium_6, Quinella, Ruminococcaceae UCG_004, Ruminococcaceae UCG_005, Allobaculum, Candidatus Saccharimonas, Ruminococcaceae UCG_010, Ruminococcus_1, Ruminococcaceae UCG_013, Ruminococcaceae UCG_014, Erysipelatoclostridium, Dubosiella, Ruminococcus_2, Eubacterium coprostanoligenes group, Parasutterella, Escherichia Shigella*, and *Anaeroplasma* at the genus level (Revised Figure 9).

The *Akkermansia* was negatively correlated with *Odoribacter, Prevotellaceae NK3B31 group, Prevotellaceae UCG_001, Alistipes, Helicobacter, Lactobacillus, Candidatus Arthromitus, A2, Blautia, GCA_900066575, Lachnospiraceae UCG_006, Eubacterium oxidoreducens group, Angelakisella, Butyricicoccus, Harryflintia, Ruminiclostridium, Ruminiclostridium_9, Ruminococcaceae NK4A214 group*, and *Desulfovibrio* at the genus level (Revised Figure 9).

## Discussion

Abdominal aortic aneurysms is a life-threatening disease, and there are still no drugs to treat it in clinical practice (Raffort et al., 2020). The risk factors for AAA included age, smoking, gender, and family history (Clancy et al., 2019). Symptoms associated with AAA may include abdominal or back pain, thromboembolism, atherosclerotic embolism, aortic rupture, or the development of an arteriovenous or aortic fistula (Ullery et al., 2018). Currently, early screening and treatment for AAA disease include one-time abdominal ultrasound screening and conventional open surgical repair or intravascular aortic stent implantation repair (Calero and Illig, 2016), but there is still a lack of effective adjuvant drugs. Studies of animal model construction of AAA showed that measurements of the aortic cross-section confirmed dilation of the lumen in AAA tissue, as well as thinning of the intermediate layer and enlargement of the outer membrane, and degeneration and destruction of the elastic layer (Yue et al., 2020). Matrix metalloproteinases (MMPs) were associated with the pathogenesis of aortic aneurysms because the histological characteristics of thoracic aortic aneurysms and AAA were loss of smooth muscle cells in the aortic middle membrane and destruction of the extracellular matrix (ECM) (Rabkin, 2017). Classic features of human AAA include ECM degeneration, MMP upregulation, and macrophage infiltration (Krishna et al., 2020). Clinical studies have shown that cathepsin is associated with collagen I degradation and inflammatory cells, and the mRNA levels of cathepsin B, D, L, and S were significantly increased in AAA compared with control aorta (Klaus et al., 2018). All these studies are consistent with the model characterization of mice in our AAA group, proving that our constructed AAA mice were consistent with the clinical characterization of AAA disease.

The role of probiotics *A. muciniphila* is known to include metabolic regulation, immune regulation, and intestinal health protection, and its abundance has been associated with various diseases such as metabolic syndrome and autoimmune diseases (Zhai et al., 2019). Recent studies have reported that *A. muciniphila* can play an important role in immunotherapy (Chen et al., 2020; Shi et al., 2020; Luo et al., 2021). Metagenomics based on stool samples from cancer patients at the time of diagnosis reveals a correlation between clinical response to immune checkpoint inhibitors and the relative abundance of *A. muciniphila* recruitment of CCR9 + CXCR3 + CD4 + T lymphocytes into the mouse tumor bed (Routy et al., 2018). *A. muciniphila* was an intestinal symbiotic bacterium with anti-inflammatory properties in the intestine and other organs, which enhanced intestinal barrier function and increased IL-10 release stimulated by whole blood lipopolysaccharide *in vitro* (Hiippala et al., 2018). *A. muciniphila* also affected the composition of immune cells in mesenteric lymph nodes, which could be seen from the increased total B cell population while reducing the total T cell and neutrophil populations (Katiraei et al., 2020). In this study, the oral administration of *A. muciniphila* in AAA mice showed that the aneurysm formation of abdominal aorta tissue was significantly inhibited, and the intestinal flora diversity and function of AAA mice were restored. Our study confirmed that *A. muciniphila* could inhibit aneurysm formation during the occurrence of AAA disease, but the specific mechanism of action still needs to be studied.

IL-33 is a member of a recently described family of IL-1 cytokines with multipotent pro-inflammatory and anti-inflammatory effects (Miller, 2011). Unlike most other cytokines, IL-33 was not secreted and was expressed constitutively in the nucleus of different cell types, where it functions as a chromatin-associated nuclear cytokine (Liew et al., 2016). During cell injury, IL-33 was released into the extracellular space and interacted with its receptor ST2, which was mainly expressed in Th2 cells, Treg cells, macrophages, and neutrophils, leading to the formation of heterodimer signaling complexes (De la Fuente et al., 2015; Tsai and Xian, 2019). The complexes included the connector protein myeloid differentiation primary reactive protein 88 (MyD88) and IL-1 receptor helper protein (IL1RAP), leading to the activation of the MAPK and NF-κB pathways (De la Fuente et al., 2015; Tsai and Xian, 2019). Less is known about IL-33 cross talk with the intestinal flora, but some studies suggest a potential interaction. Studies conducted in SAMP mice have shown that transplantation of common feces induces IL-33 expression in ex-GF mice (De Salvo et al., 2016). Furthermore, IL-33 expression had many known interactions with microbial-sensing Toll-like receptors (TLRs), and was indeed induced by pathogen-associated molecular patterns (PAMPs), perhaps as a protective mechanism in the host (Natarajan et al., 2016). Our study found that the expression of IL-33 was increased in the abdominal aorta tissues of AAA mice, and the expression of IL-33 was decreased after treatment with *A. muciniphila*. We speculated that the inhibitory effect of *A. muciniphila* on the formation of abdominal aortic aneurysms might be related to the molecular immunity of distal IL-33 through gut microbiota, but it still needs further study. It is unknown whether the Akk-reduced lesion was caused by an increase in bacterial diversity or Akk-reduced IL-33. In addition, we have not found a suitable method for quantitative analysis of *A. muciniphila* in the fecal sample except for the 16S rRNA gene sequencing. This is the limitation of our study. We also failed to show a cause-effect relationship, such as the reconstitution of IL-33-reversed Akk-reduced lesion. The highly personalized microbiome and its complex and multidirectional interactions with host metabolism and immunity present potential opportunities for the development of the next generation of microbiome-based drugs and diagnostic biomarkers (Mousa et al., 2022). We will continue to explore this in the subsequent scientific research.

*Lactobacillus casei* cell wall extract (LCWE) induced systemic arterial dilation and aneurysm development, including AAA, in a mouse model of Kawasaki disease (KD) vasculitis (Wakita et al., 2016). Both IL-1α and IL-1β played a key role in KD disease, and the use of IL-1R blockers that inhibited both pathways might be a therapeutic target not only for KD coronary arteritis but also for other systemic aneurysms, including AAA (Lee et al., 2015; Wakita et al., 2016). There was a study that showed species from *Akkermansia muciniphila* and *Lachnospiraceae bacterium A2* were significantly higher in the control group than that in the AAA group, while six species, namely, *Lachnospiraceae bacterium, COE1, Corynebacterium stationis, Firmicutes Bacterium ASF500, Helicobacter bilis*, and *Clostridium leptum*, were increased in the AAA group (Xie et al., 2020). Our study found that Firmicutes-*Lactobacillus* increased in AAA mice and decreased after treatment with *A. muciniphila*. In addition, the effects of *Helicobacter pylori* and *Lactobacillus acidophilus* on peripheral blood mononuclear cell (PBMC) cytokine profiles showed changes in IL-10, Th1, and Th2 cytokines, but there were inherent differences between patients and healthy people (Aria et al., 2020). In addition, *Helicobacter pylori* and *Lactobacillus acidophilus* also affected the changes in miRNA expression profiles in CD4 + memory T cells in AAA patients and healthy controls, participating in the development and prevention of AAA (Kalani et al., 2021). In our study, we found that the *Akkermansia* was negatively correlated with *Helicobacter* and *Lactobacillus* at the genus level. These studies suggest that the mechanism of *A. muciniphila* inhibiting AAA formation might be related to the changes in the abundance of intestinal *Lactobacillus*.

L-Rhamnose could be used by the thermophilic bacterium *Clostridium stercorarium* to produce D-allose from D-allulose (Seo et al., 2018). L-Rhamnose could also be used as a carbon source and energy source for the growth of *E. coli* (Chen and Lin, 1984). L-Rhamnose inhibited phage adsorption to cells without inactivating free phages (Watanabe and Takesue, 1975). The inhibition of L-rhamnose on phage adsorption was competitive against host cells (Watanabe and Takesue, 1975). The function prediction based on the MetaCyc database showed the superpathway of fucose and rhamnose degradation (LDA = 3.00, *P* = 0.007) and L-rhamnose degradation I (LDA = 2.88, *P* = 0.021) pathways were enriched in the control group, whereas the dTDP-L-rhamnose biosynthesis I (LDA = 2.91, *P* = 0.004) pathway was enriched in the AAA group. In addition, the degradation of L-rhamnose in the intestinal microbiota metagenomic functional pathway during immunotherapy in melanoma patients was associated with progression-free survival (Peters et al., 2019). In our study, we found that the *Akkermansia* was positively correlated with *Clostridium sensu stricto_1* and *Escherichia shigella* at genus level. The above studies have proved that intestinal microbial-related L-rhamnose degradation and biosynthesis pathways are reversed in AAA disease, which may be the target of *A. muciniphila* to regulate the function of gut microbiota.

## Conclusion

The occurrence of AAA disease might be accompanied by increased expression of inflammatory cytokines, decreased gut microbiota diversity, severe local immune infiltration, and elastic fiber degradation in the abdominal aorta. Oral administration of *A. muciniphila* could inhibit the expansion and inflammation of the abdominal aorta, restore the gut microbiota diversity, promote peripheral blood immunity and IL-33 expression, and regulate the functional pathways related to *Lactobacillus* and L-rhamnose degradation/synthesis to inhibit the formation of AAA.

## Data Availability Statement

The datasets presented in this study can be found in online repositories. The names of the repository/repositories and accession number(s) can be found in the article/supplementary material.

## Ethics Statement

This study was approved by the Medical Ethics Committee of Xiangya Hospital of Central South University (202104809), Changsha, Hunan, China.

## Author Contributions

KW and XH designed and performed the study. YB and HZ analyzed the data. All authors contributed to the writing and revisions and reviewed the manuscript.

## Conflict of Interest

The authors declare that the research was conducted in the absence of any commercial or financial relationships that could be construed as a potential conflict of interest.

## Publisher’s Note

All claims expressed in this article are solely those of the authors and do not necessarily represent those of their affiliated organizations, or those of the publisher, the editors and the reviewers. Any product that may be evaluated in this article, or claim that may be made by its manufacturer, is not guaranteed or endorsed by the publisher.

## Footnotes

1
http://www.bioinformatics.com.cn/static/others/jvenn/example.html

2
https://github.com/picrust/picrust2

3
https://metacyc.org/

4
https://github.com/SegataLab/lefse
